# Hormonal contraceptive use and the risk of sexually transmitted infections: a systematic review and meta-analysis

**DOI:** 10.1038/s41598-022-24601-y

**Published:** 2022-11-25

**Authors:** Tasnima Akter, Mario Festin, Angela Dawson

**Affiliations:** 1 School of Public Health, Faculty of Health, University of Technology Sydney, Sydney, Australia; 2 College of Medicine, University of the Philippines, Manila, Philippines

**Keywords:** Disease prevention, Health care, Public health, Epidemiology

## Abstract

There are 150 million women worldwide using combined or progestogen-only hormonal contraceptive methods who may be at risk of sexually transmitted infections (STIs). Previous systematic reviews that have sought to establish whether there is an aetiological association between hormonal contraceptive methods/use and STIs have been limited in their methods and have mixed findings. We sought to update these reviews using appropriate control groups. We undertook a systematic review following the PRISMA guidelines and meta-analysis to examine the association between the use of all hormonal contraceptive methods and the acquisition of STIs (Neisseria gonorrhoeae, syphilis/Treponema pallidum, Chlamydia trachomatis, herpes simplex virus, and Trichomonas vaginalis) and/or bacterial vaginosis in literature published between 2005 and 2020. We analysed the effect of hormonal contraceptive methods/use separately on the prevalence, incidence and recurrence of STIs. A total of 37 studies were included in this review that reported 61 associations, in which 27 prevalence, eight incidence and two recurrence studies provided 43, 16, and two associations, respectively. We observed a positive association between hormonal contraceptive methods/use and the risk of chlamydia and herpes but a negative association for trichomoniasis and vaginosis. A negative but statistically insignificant association was observed between hormonal contraceptive methods/use and gonorrhoea. Hormonal contraceptive methods/use influences a woman's risk of STIs/ bacterial vaginosis, but the risk may differ depending on the type of STI. These findings should be contextualized carefully, particularly when formulating practice guidelines and policy, as the effects of hormonal contraceptive methods/use on the risk of STIs varied in direction when analysed separately by STI.

## Introduction

Globally the acquisition of new sexually transmitted infections (STIs) is very high—around 357 million per year^1^. Specifically, there are approximately 131 million cases of chlamydia trachomatis (CT), 78 million cases of Neisseria gonorrhoeae (NG), 143 million cases of trichomonas vaginalis (TV) and 5.6 million cases of syphilis/Treponema pallidum (ST)^1^. The prevalence of viral STIs is also high. Approximately 500 million cases of herpes simplex virus (HSV) are recorded each year and there are 290 million cases of women with human papillomavirus (HPV)^1^. The high incidence of STIs has an adverse effect on sexual and reproductive health. STIs may significantly increase the risk of human immunodeficiency virus (HIV) acquisition, cause cervical cancer and lead to pelvic inflammatory disease (PID), infertility, ectopic pregnancy, miscarriage, fetal death, stillbirth, neonatal death, and congenital infections^1^.

Hormonal contraceptives (HC) are used worldwide by more than 150 million women and therefore, an investigation into whether there is an aetiological association between STIs and HC is an important question for public health^2^. HC that includes combined oestrogen-progesterone contraception (e.g. oral contraceptive pills), progestin-only injectables (e.g. depo-medroxyprogesterone acetate), and hormone-containing technologies (e.g. implants, intrauterine devices) reduce unwanted pregnancy and maternal morbidity and mortality. However, previous systematic reviews have reported that HC may increase the risk of HIV acquisition, CT and HSV type 2^3–7^. In contrast, some reviews have found an inconclusive effect of HC on the incidence of NG and TV, while other reviews have observed that HC can reduce the risk of TV and bacterial vaginosis (BV)^5–10^.

Previous reviews examining the association between HC and the risk of STIs are outdated and/or did not include a meta-analysis of data, or used comparisons of groups that included HC users. For example, reviews by Mohllajee et al.^6^ and Morrison et al.^7^ include studies up to 2008. On the other hand, reviews by Vodstrcil et al.^9^ and Van de Wijgert et al.^8^ were limited to specific STIs and/or considered control groups that also included users of HC. Furthermore, reviews by McCarthy et al.^5^, Deese et al.^10^, Van de Wijgert et al.^8^, Mohllajee et al.^6^ and Morrison et al.^7^ did not include a meta-analysis. A clear understanding of the association between hormonal contraceptive use (HC-use) and the risk of STIs requires a meta-analysis to draw accurate conclusions based on studies that used appropriate control groups.

An updated review of the association of HC-use with the risk of STIs may help to provide insights to improve infection control and consequently achieve the health-related targets of the Sustainable Development Goal 3 (SDG3)^11,12^. Specifically, such a study may also contribute to ending the preventable deaths of newborns and children under five years (SDG3 target 2), combating the epidemics of AIDS and other communicable diseases (SDG3 target 3) and ensuring universal access to sexual and reproductive health care (SDG3 target 7)^11,12^. Therefore, this systematic review and meta-analysis aimed to examine the association between the use of HC and the acquisition of STIs based on the literature published between 2005 and 2020.

## Methods

The Preferred Reporting Items for Systematic Reviews and Meta-Analyses (PRISMA) guideline was used to conduct this systematic review and meta-analysis^13^ and is registered with PROSPERO, CRD42021272742.

### Search protocol

#### Inclusion criteria

A Population, Interventions, Comparators, Outcomes, Study design (PICOS) question, as suggested in the Centre for Reviews and Dissemination (CRD) CRD^14^, was formed to lead this review. Thus, this review considered women of reproductive age (15–49 years) as participants. Specifically, we focused on studies involving HC-use by women of reproductive age and tested for prevalence (a measure of a condition in a population at a given point/period), incidence (number of new occurrences of a condition in a population over a period), or recurrence (return of a condition after a remission) of STIs using standard diagnostic methods. This review considered all HC methods as interventions/exposures and included combined oestrogen-progesterone contraception (e.g., combined oral contraceptive pills, combined contraceptive patch, transdermal patch, ortho evra, combined injectable contraceptive, combined contraceptive vaginal ring, vaginal ring, nuva ring, emergency contraceptive pill), progesterone-only contraception (e.g., progesterone-releasing vaginal ring, proge-ring, depot medroxyprogesterone acetate, intrauterine device, levonorgestrel), as well as the use of unspecified HC. The comparator/control group was the non-users of HC (i.e., women who did not use any contraceptives or used non-HC). The outcome of interest was the following STIs: Neisserria gonorrhoeae, Syphilis/Treponema pallidum, Chlamydia Trachomatis, Herpes Simplex Virus, and Trichomonas Vaginalis. We also considered Bacterial Vaginosis and Pelvic Inflammatory Disease as our outcome of interest. HIV and HPV were not included as there is already an updated review in relation to HIV^15^ and HPV is beyond the scope of our study. Randomised trials (with either individual or cluster allocation), observational and descriptive studies were eligible for inclusion in this review.

#### Exclusion criteria

This investigation excluded review articles, and studies that did not use standard methods to diagnose STIs, had no comparison groups and did not investigate the association of hormonal contraception with STIs. Following Vodstrcil et al.^9^ we excluded studies reporting either less than 20 STIs cases or less than 10% of participants exposed to HC.

### Search strategy

We focused on studies on HC and STIs and searched PubMed and EMBASE databases for relevant peer-reviewed articles published between 2005 and 2020 and written in English. Following the other reviews, we selected a 15-year period to ensure an analysis of the most contemporary research^16,17^. The Medline MeSH subject headings employed in this review were: *hormonal contraceptive* OR *contraceptive* OR *contraception* OR *Combined oral Contraceptive* OR *Oral Contraceptive* OR *oestrogen* OR *Progesterone-releasing vaginal ring* OR *Progering* OR *progesterone* OR *progestin* OR *Intrauterine device* OR *depot medroxyprogesterone acetate* OR *DMPA* OR *Emergency contraceptive* OR *Levonorgestrel* AND *Sexually Transmitted Diseases* OR *Sexually Transmitted Infection* OR *STI* OR *Gonorrhoea* OR *Chlamydia Trachomatis* OR *Herpesvirus 1, Human* OR *Herpesvirus 2, Human* OR *Trichomonas Vaginitis* OR *Trichomonas Vaginalis* OR *Bacterial Vaginosis* OR *Bacterial Vaginitis* OR *Pelvic Inflammatory Disease* OR *Syphilis*.

**Figure 1:**
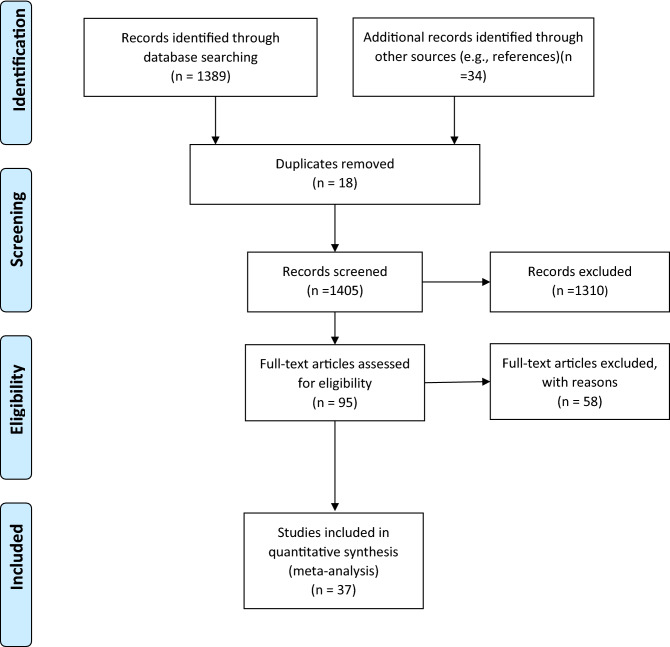
Literature review process.

We initially retrieved 1,405 articles and screened these articles as per the PICOS question (Fig. 1). The screening process retained 95 articles, which were examined in more detail. At this stage, studies were excluded because of < 20 cases of STIs (n = 6), use of non-standard diagnostic methods (n = 3), < 10% of the study population using HC (n = 2), did not mention the proportion of participants using HC (n = 1), not satisfying the outcome of interest (n = 15), inappropriate control groups (n = 27) and not analysing the association between STIs and HC (n = 4). Thus, a total of 37 articles were selected for the quality assessment.

### Data extraction and synthesis

From each included study, data were extracted systematically on: the author(s); analysis country; study design; sample size; the participants' characteristics; outcomes (the type of STIs/BV/PID); STI/BV/PID outcome measure (prevalence/incidence/recurrence); the number of participants with STIs; the diagnostic method employed; the exposures (the type of HC method used, the proportion of women using HC); the comparison group (hormonal users vs. non-users); the type of statistical analysis performed; the control variables used in the adjusted analysis (e.g., condom use; whether the study has included information on participants' HIV seropositivity); the size of the estimate, and the significance level.

### Risk of bias (quality) assessment

Following the previous literature, existing quality assessment tools—MOOSE, STROBE and QATSO—were used to evaluate the quality of the selected studies before the initiation of data synthesis^9,18–20^. The risks of bias in studies were assessed regarding the selection of participants, study design, confounders and analyses. Other potential risks of bias included whether the study had reported a standard method of STIs assessment and clearly defined the HC-use.

### Statistical analyses

The primary objective of this study was to examine the association between HC-use and STIs/BV/PID. We directly copied both the estimates and the 95% confidence intervals from the selected studies. In the case of their unavailability, we calculated those numbers using raw data from those studies. Then, we undertook a meta-analysis using either fixed or random effects models depending on the I^2^ test for heterogeneity as described in Vodstrcil et al.^9^. A meta-analysis based on a fixed-effect model was conducted if the I^2^ statistic was < 25% and used a random-effect model if the I^2^ statistic was between 25 and 75%, otherwise, the studies were not analysed. We preferred adjusted over unadjusted results, in case both were available for any study, as the former netted out the effect of confounders.

The analysis initially investigated the effect of HC-use on composite STIs outcome measures (prevalence, incidence and recurrence of STIs). In that analysis, following Vodstrcil et al.^9^, we attempted to convert the odds ratios (ORs) to risk ratios (RRs) in studies where data was available. Then, we analysed the effect of HC-use separately on prevalence, incidence and recurrence of STIs. All of these analyses were stratified by types of STIs/BV/PID, as described earlier.

Stata version 15 was used for all the analyses.

### Sensitivity analyses

Following the previous research^9^, we conducted several sensitivity checks where we excluded some specific sub-groups to check the robustness of our results. The large sample size allowed us to conduct the analysis by omitting studies in which all women were sex workers or studies including HIV-positive participants since these groups are more vulnerable to develop/spread STIs; or studies in which comparison group contained women not using any contraception; or studies in which women were selected based on RCT design.

### Risk of bias across studies

We used funnel plots to assess the potential presence of publication bias in studies reporting the prevalence and incidence of STIs, as suggested in Vodstrcil et al.^9^. Asymmetry was statistically evaluated using the Egger's correlation tests by regressing the log of the estimate (unadjusted/adjusted OR or RR) on the log of the standard error (SE) of the estimate. The SEs were estimated based on the width of the reported confidence interval using the formula (ln[upper limit of CI]-ln[OR or RR])/1.96.

## Results

All 37 studies in this review are summarized in Table 1. Among these studies, 14 were carried out in the United States of America, 14 in Africa, two in Europe, three in Australia and four in Asia. The majority of the studies (16) were based on cross-sectional designs. The other design used in the selected studies were longitudinal cohort (five), prospective cohort (eight), randomised controlled trial (four), retrospective cohort (two) and case–control (two). Some studies separately investigated the association between STIs and different types of HC-use (or, vice versa) and, when a study provided more than one association, we chose those, which persuade the inclusion criteria of this review (Tables 1 and 2). As a result of this process, 37 studies were identified reporting 61 associations, in which 27 prevalence, eight incidence and two recurrence studies provided 43, 16 and two associations, respectively. While summarizing these studies, we also observed 13 associations attributed to the combined oestrogen-progesterone contraception (COC), 32 investigated progesterone-only contraception (POC) and 16 association provided for unspecified hormonal contraception (UHC).

**Table 1:** Characteristics of prospective studies included in the systematic review.

Reference	Study country	Study design	Outcome measure	HC-type used	Diagnostic method	Sample size	% positive	% using HC	HC-use comparison group	Unadjusted OR/RR (95% CI)	Reported^a^ adjusted OR/RR (95% CI)
***Neiserria gonorrhoea* (n = 6)**											
Kleinschmidt et al.^21^	South Africa	CS	Prevalence	POC	Nugent	551	3.8	54.4	NHC/NC	1.37 (0.56–3.37)	
Pettifor et al.^22^	South Africa	LC	Incidence	POC	Ligase chain reaction	567	4.0	19.9	NHC/NC	1.19 (0.53–2.65)^b^	1.30 (0.58–2.98)
				POC	Ligase chain reaction	567	4.0	34.7	NHC/NC	1.41 (0.70–2.85)^b^	1.11 (0.55–2.25)
Gursahaney et al.^23^	USA	LC	Prevalence	COC	Gram stain, oxidase testing and Gonochek II analysis	107	63.6	21.0	NHC/NC	0.42 (0.22–0.78)^b^	0.43 (0.23–0.81)
Hancock et al.^24^	USA	CS	Prevalence	UHC	Gram staining and/or culture	1,090	2.4	35.7	NHC/NC		0.20 (0.05–0.58)
Wand and Ramjee^25^	South Africa	PC	Prevalence	POC	Nucleic acid amplification assay	2,236	22.0	46.5	NHC	1.28 (0.69–2.40)^b^	1.31 (0.69–2.50)
Borgdorff et al.^26^	Rwanda	PC	Prevalence	POC	Amplicor CT/NG PCR test	800	5.8	12.1	NHC/NC		1.13 (0.47–2.77)
***Chlamydia trachomatis* (n = 7)**											
Kleinschmidt et al.^21^	South Africa	CS	Prevalence	POC	Nugent	551	13.8	54.4	NHC/NC	0.97 (0.60–1.57)	
Pettifor et al.^22^	South Africa	LC	Incidence	POC	Ligase chain reaction	567	14.0	19.9	NHC/NC	1.18 (0.77–1.81)^b^	1.24 (0.80–1.94)
				POC	Ligase chain reaction	567	14.0	34.7	NHC/NC	0.93 (0.60–1.43)^b^	0.91 (0.59–1.43)
Tibaldi et al.^27^	Italy	CS	Prevalence	COC	Transcription mediated amplification	27,172	1.4	15.2	NC	1.68 (1.09–2.59)^b^	1.51 (0.93–2.47)
Wand and Ramjee^25^	South Africa	PC	Prevalence	POC	Nucleic acid amplification assay	2,236	22.0	46.5	NHC	2.22 (1.39–3.52)^b^	2.46 (1.52–3.97)
Masese et al.^28^	Kenya	PC	Incidence	POC	Gen-probe aptima	865	5.0/100^g^	20.9	NHC/NC	1.90 (1.10–3.20)^b^	1.80 (1.10–3.00)
Forcey et al.^29^	Australia	CS	Prevalence	UHC	Nucleic acid amplification	5,055	5.8	39.2	NHC/NC	1.80 (1.40–2.30)^b^	1.60 (1.30–2.00)
Borgdorff et al.^26^	Rwanda	PC	Prevalence	POC	Amplicor CT/NG PCR test	800	2.5	12.1	NHC/NC		1.96 (0.59–6.57)
**Syphilis-treponemapallidum (n = 1)**											
Borgdorff et al.^26^	Rwanda	PC	Prevalence	POC	Spinreact Rapid Plasma Reagin test with confirmation by Spinreact T. pallidum Haemagglutination test	800	3.6	12.1	NHC/NC		2.22 (0.82–6.05)
***Trichomonas vaginalis* (n = 9)**											
Kleinschmidt et al.^21^	South Africa	CS	Prevalence	POC	Nugent	551	7.4	54.4	NHC/NC	0.78 (0.41–1.48)	
Pettifor et al.^22^	South Africa	LC	Incidence	POC	Diamond	567	7.8	19.9	NHC/NC	0.38 (0.13–1.08)^b^	0.35 (0.12–1.01)
				POC	Diamond	567	7.8	34.7	NHC/NC	0.59 (0.28–1.23)^b^	0.63 (0.30–1.29)
Tibaldi et al.^27^	Italy	CS	Prevalence	COC	Microscopy	27,172	1.6	15.2	NC	0.53 (0.30–0.94)^b^	0.56 (0.29–1.08)
Torok et al.^30^	USA	CC	Prevalence	COC	InPouch culture system	571	74.8	14.0	NHC/NC	0.50 (0.30–0.80)^b^	0.90 (0.50–1.60)
Baris and Arman Karakaya^31^	Turkey	PC	Prevalence	COC	Pap-stained smear samples—Bethesda 2001 criteria	638	4.9	16.8	NHC/NC	0.52(0.16–1.74)	
Huppert et al.^32^	USA	CS	Prevalence	UHC	Nucleic acid amplification testing	215	24.0	35.8	NHC/NC	0.77(0.40–1.49)	
Brahmbhatt et al.^33^	Uganda	PC	Incidence	POC	InPouch test	2,374	2.4/100^g^	28.0	NHC/no condom	0.53 (0.30–0.95)^b^	0.54 (0.30–0.98)
Borgdorff et al.^26^	Rwanda	PC	Prevalence	POC	Wet mount or InPouch test	800	9.4	12.1	NHC/NC		0.77 (0.32–1.83)
Ngcapu et al.^34^	South Africa	PC	Prevalence	POC	PCR	128	18.9	50.0	NHC/NC	1.00(0.41–2.43)	
**Pelvic inflammatory disease (n = 1)**											
Berenson et al.^35^	USA	RC	Incidence	POC	ICD-9	90,489	0.08	78.0	Copper IUD		0.68 (0.53, 0.86)
**Herpes simplex virus type 2 (n = 3)**											
Kenyon et al.^36^	South Africa	CS	Prevalence	UHC	Enzyme-linked immunoassay	784	53.3	54.2	NHC/NC	1.40 (1.00–1.94)^b^	1.70 (1.10–2.60)
Borgdorff et al.^26^	Rwanda	PC	Prevalence	POC	HerpeSelect 2 ELISA	800	60.6	12.1	NHC/NC		2.13 (1.26–3.59)
Grabowski et al.^37^	Uganda	PC	Incidence	POC	ELISA	682	10.0	13.5/100^g^	NHC/NC	2.02 (0.96–4.26)^b^	2.26 (1.09–4.69)
**Bacterial vaginoses (n = 25)**											
Ashraf Ganjoei^38^	Iran	CS	Prevalence	COC	Amsel	130	37.7	NA^d^	NHC/NC	0.37 (0.14–0.99)^b^	
Bradshaw et al.^39^	Australia	CC	Prevalence	COC	Amsel/Nugent	342	46.0	46.8	NHC/NC	0.60 (0.40–0.90)^b^	0.60 (0.40–1.00)
Harville et al.^40^	USA	CS	Prevalence	UHC	Nugent	411	26.0	42.1	NHC/NC	0.61(0.38–0.96)	
Schwebke and Desmond^41^	USA	LC	Incidence	UHC	Nugent	96	69.8	41.7	NHC/NC	0.81 (0.61–1.08)	
Bradshaw et al.^39^	Australia	LC	Recurrence	UHC	Nugent	139	58.0	38.1	NHC/NC	0.40 (0.20–0.80)^b^	0.50 (0.30–1.00)
Amaral et al.^42^	Brazil	CS	Prevalence	UHC	Nugent	155	75.5	44.5^c^	NHC/NC	0.56 (0.25–1.26)^c^	
Evans et al.^43^	UK	CS	Prevalence	UHC	Ison-Hay	189	14.4	51.0	NHC/NC	0.77 (0.30–1.98)^b^	
Kleinschmidt et al.^21^	South Africa	CS	Prevalence	POC	Nugent	554	34.7	54.4	NHC/NC	0.96 (0.68–1.36)	
Cherpes et al.^44^	USA	LC	Incidence	COC	Nugent	773	36/100^g^	62.9^gc^	NC	0.80 (0.60–1.10)^b^	
				POC	Nugent	773	36/100^g^	280.7^gc^	NC	1.20 (0.80–1.90)^b^	
McClelland et al.^45^	Kenya	RCT/LC	Incidence	POC	Nugent	151	37.1	28.5	NC/TL	0.59 (0.48–0.73)^b^	0.60 (0.48–0.74)
Peipert et al.^46^	USA	RCT/CS	Prevalence	UHC	Amsel/Nugent	523	31.0	32.0	NHC/NC	0.70 (0.46–1.05)	
Baisley et al.^47^	Tanzania	CS	Prevalence	UHC	Nugent	1,305	62.9	30.0	NHC/NC	0.72 (0.56–0.92)^b^	0.80 (0.62–1.04)
Pettifor et al.^22^	South Africa	LC	Incidence	POC	Nugent	567	35.6	19.9	NHC/NC	0.75 (0.55–1.02)^b^	0.77 (0.56–1.06)
				POC	Nugent	567	35.6	34.7	NHC/NC	0.89 (0.69–1.14)^b^	0.91 (0.70–1.18)
Rifkin et al.^48^	USA	CS	Prevalence	COC	Amsel	330	40.3	58.2	NHC	1.01 (0.67–1.52)^b^	0.66 (0.39–1.10)
				POC	Amsel	330	40.3	17.0	NHC	0.42 (0.24–0.74)^b^	0.42 (0.20–0.88)
Tibaldi et al.^27^	Italy	CS	Prevalence	COC	Amsel	27,172	8.9	15.2	NC	0.86 (0.72–1.04)^b^	0.69 (0.56–0.85)
Yotebieng et al.^49^	Thailand	RCT/CS	Prevalence	UHC	Amsel	901	57.0	24.9	NHC/NC	0.46 (0.34–0.63)	
Brotman et al.^50^	USA	CS	Prevalence	UHC	Amsel	93	67.0	12.9	NHC/NC	1.00 (0.28–3.62)^b^	
Bukusi et al.^51^	Kenya	RCT/LC	Recurrence	UHC	Nugent	164	42.7	33.5	NHC/NC	1.11 (0.77–1.60)^c^	
Kampan et al.^52^	Malaysia	CS	Prevalence	UHC	Amsel	131	19.1	49.6	NHC/NC	0.86 (0.32–2.23)^c^	
Mascarenhas et al.^53^	Brazil	CS	Prevalence	UHC	Nugent	100	20.0	41.0	NHC/NC	0.95 (0.35–2.58)	
Baris and Arman Karakaya^31^	Turkey	PC	Prevalence	COC	Pap-stained smear samples—Bethesda 2001 criteria	638	9.4	16.8	NHC/NC	0.74(0.34–1.62)	
Jespers et al.^54^	Sub-Saharan Africa	CS	Prevalence	POC	Nugent	430	36.0	33.3	NHC/NC	0.85 (0.53–1.37)^b^	
				COC	Nugent	430	36.0	34.8	NHC/NC	0.91 (0.47–1.76)^b^	
Ngcapu et al.^34^	South Africa	PC	Prevalence	POC	Nugent	128	51.2	50.0	NHC/NC	0.94 (0.47–1.88)	
Francis et al.^55^	Uganda	PC	Prevalence	POC	Nugent	1027	56.0	24.4	NC	0.63 (0.47–0.85)^b^	0.66 (0.50–0.86)
Brooks et al.^56^	USA	RC	Prevalence	COC	Amsel's criteria	682	12.0	30.0	Condom		0.29 (0.13–0.64)
				POC	Amsel's criteria	682	12.0	13.7	Condom		0.34 (0.13–0.89)
				POC	Amsel's criteria	682	12.0	28.7	Condom		1.55 (0.72–3.35)

^a^All adjusted OR/RRs are reported by authors.

^b^OR/RRs reported by authors.

^c^Estimation recorded from Vodstrcil et al.^9^.

^d^Raw data on the % using OC not available, on the basis of the odds ratios reported, the proportion of women using contraceptives were calculated to well exceed 10%

^g^Woman-years, calculated by authors.

*LC* Longitudinal cohort, *PC* Prospective cohort, *CS* Cross sectional, *RCT* Randomised controlled trial, *RC* Retrospective cohort, *CC* Case control.

*NHC* No hormonal contraception, *NC* No contraception, *POC* progesterone-only containing methods of hormonal contraception, *COC* Combined oestrogen- and progesterone containing methods of hormonal contraception, *UHC* Unspecified hormonal contraception, *TL* Tubal ligation.

**Table 2:** Assessment of bias: measures of the studies included in the analysis.

Measure	Variables	N-studies/associations
STIs outcome measure (37 studies)	Prevalence	27^a^
	Incidence	8^a^
	Recurrence	2^a^
Hormonal contraceptive type (37 studies, 61 associations)	COC	13^a^
	POC	32^a^
	UHC	16^a^
Settings/recruitment venue (37 studies)	Sexual or reproductive health service	24^b^
	General community healthcare service	3^c^
	Sex worker Service	6^d^
	Population based	4^e^
HC-use comparison group (61 associations)	No contraceptive use	6^a^
	No hormonal contraceptives use	8^a^
	No hormonal contraceptives use or no contraceptive use	47^a^
Reported outcome estimates (61 associations)	Adjusted analyses Condom used as adjusted variable	37^a^ 31^a^
	Unadjusted analyses	24^a^

^a^References provided in Table 1.

^b^Ashraf Ganjoei^38^, Bradshaw et al.^39,57^, Brotman et al.^50^, Bukusi et al.^51^, Cherpes et al.^44^, Evans et al.^43^, Forcey et al.^29^, Grabowski et al.^37^, Gursahaney et al.^23^, Hancock et al.^24^, Harville et al.^40^, Huppert et al.^32^, Jespers et al.^54^, Kampan et al.^52^, Kleinschmidt et al.^21^, Mascarenhas et al.^53^, Peipert et al.^46^, Pettifor et al.^22^, Rifkin et al.^48^, Schwebke and Desmond^41^, Tibaldi et al.^27^, Torok et al.^30^, Wand and Ramjee^25^.

^c^Baisley et al.^47^, Baris and Arman Karakaya^31^, Brooks et al.^56^.

^d^Amaral et al.^42^, Borgdorff et al.^26^, Francis et al.^55^, Masese et al.^28^, McClelland et al.^45^, Yotebieng et al.^49^.

^e^Berenson et al.^35^, Brahmbhatt et al.^33^, Kenyon et al.^36^, Ngcapu et al.^34^.

*POC* progesterone-only containing methods of hormonal contraception, *COC* Combined oestrogen- and progesterone containing methods of hormonal contraception, *UHC* Unspecified hormonal contraception.

### Risk of bias within studies

We observed that the majority of the studies under this review adequately discussed the selection criteria of participants and clearly described the study design. In terms of the study recruitment venue, 24 studies were conducted at sexual or reproductive health service setting, three at general community healthcare service setting and six at sex worker service setting, while four were population-based (Table 2). However, the sensitivity analyses of the exclusion of sex worker service settings did not show any bias in our estimation (Supplementary Fig. 1). The inclusion criteria in this review restricted us only to select studies that used standard diagnostic methods and a clear definition of exposed and control groups. Therefore, the issues with diagnostic methods and group classification were clearly addressed in the included studies. It is worth mentioning that 16 UHC-use based associations are less likely to cause bias in this review as this research focus on the use of HC, not the type. We also observed that, while the comparison groups in the selected studies were different, 47 out of 61 associations had the same control group (Table 2). Notably, 37 reported estimates were based on adjusted analyses, of which 31 adjusted for the use of condoms. However, our sensitivity analyses showed that none of these caused any bias. Furthermore, we checked publication bias using funnel plots within the individual STIs (Fig. 6) but did not find any significant indication of bias, and this is discussed in detail at the end of this result section.

### Synthesis of association between HC-use and specific STI/BV/PID

#### Neiserria Gonorrhoea

Six studies evaluated the association between HC-use and the risk of incidence or prevalence of NG^21–26^ (Table 1). Among these, two studies^23,24^ observed a reduced risk of NG among women using COC (OR 0.43; 95% CI 0.23–0.81) and UHC (OR 0.20; 95% CI 0.05–0.58), respectively.

**Figure 2:**
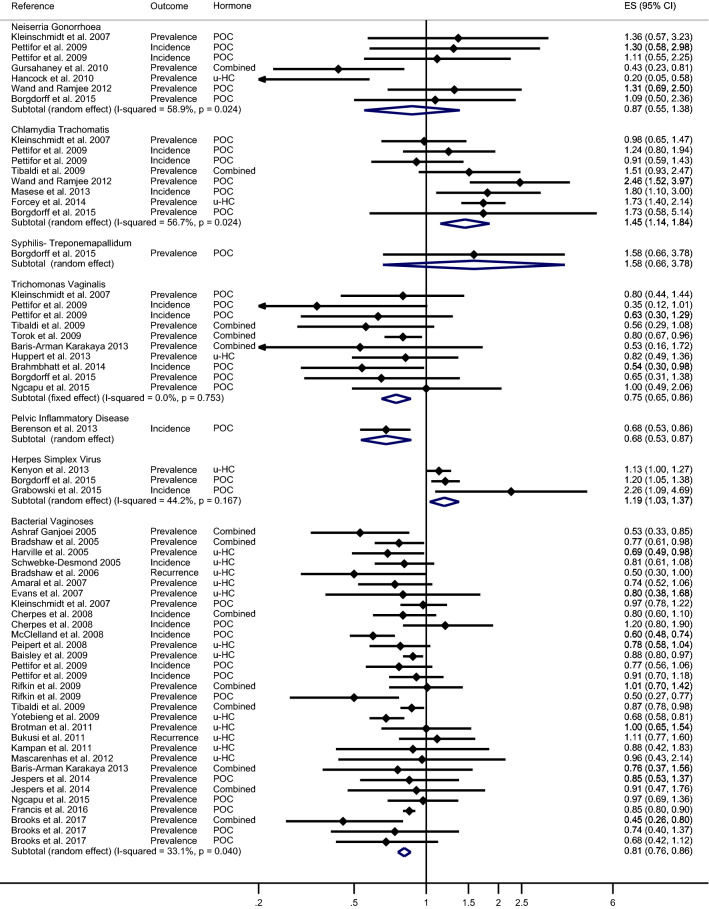
Meta-analysis of the association between hormonal contraceptives and STIs/BV outcome. The diamond in the middle of each horizontal line represents the point estimate of the effect for a single study. Each horizontal lines depict the 95% confidence interval (CI) for a study and the lines that extend beyond the specified value range are cropped and adorned with arrows. The group-specific effect size is plotted by diamond (without horizontal line) with the width corresponding to its 95% CI.

**Figure 3:**
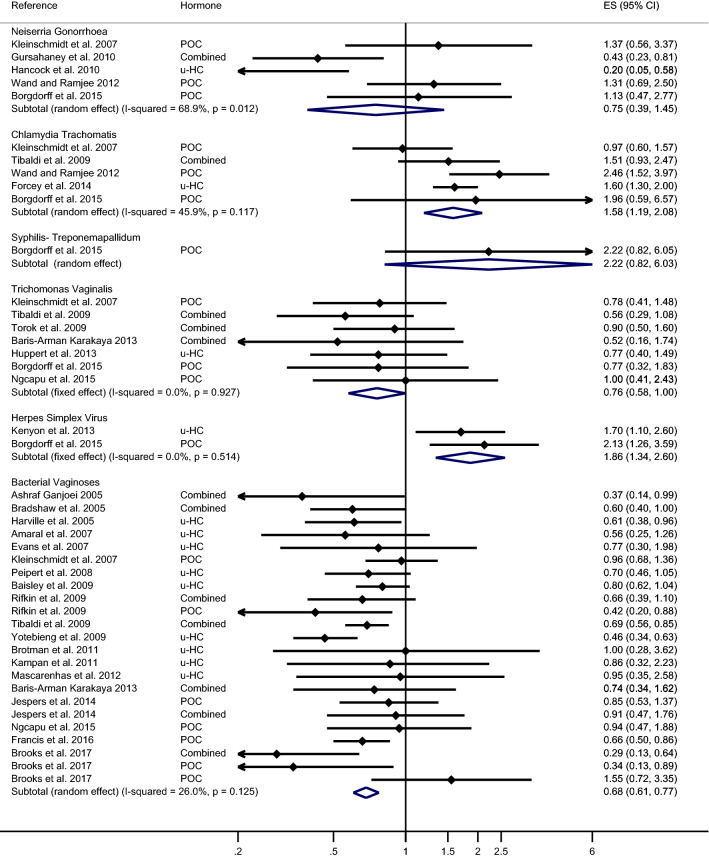
Meta-analysis of the association between hormonal contraceptives and the prevalence of STIs/BV. The diamond in the middle of each horizontal line represents the point estimate of the effect for a single study. Each horizontal lines depict the 95% confidence interval (CI) for a study and the lines that extend beyond the specified value range are cropped and adorned with arrows. The group-specific effect size is plotted by diamond (without horizontal line) with the width corresponding to its 95% CI.

**Figure 4:**
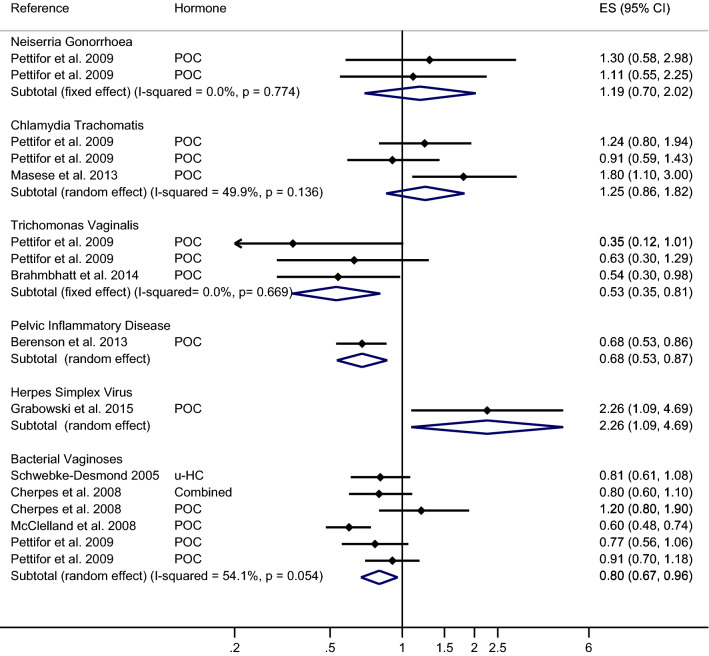
Meta-analysis of the association between hormonal contraceptives and the incidence of STIs/BV. The diamond in the middle of each horizontal line represents the point estimate of the effect for a single study. Each horizontal lines depict the 95% confidence interval (CI) for a study and the lines that extend beyond the specified value range are cropped and adorned with arrows. The group-specific effect size is plotted by diamond (without horizontal line) with the width corresponding to its 95% CI.

The overall estimates of meta-analysis demonstrated that HC-use did not significantly reduce the risk of NG in comparison with no HC-use or no contraceptive use (pooled effect size by random-effects [reES] = 0.87; 95% CI 0.55–1.38) with the heterogeneity between the studies were 59% (Fig. 2). We then conducted the stratified analysis of the prevalence and incidence of NG with HC-use (Figs. 3 and 4, respectively) and the outcome did not show any significant association (reES = 0.75; 95% CI 0.39–1.45 and feES = 1.19; 95% CI 0.70–2.02, respectively).

#### Chlamydia trachomatis

We identified seven studies that evaluated the association between HC-use and the risk of CT^21,22,25–29^ (Table 1). Of these, four studies observed no significant association, while the other three reported a significant increase in CT with the use of HC. A study by Wand and Ramjee^25^ observed that POC users were 2.46 times more likely to develop the risk of CT compared to those who did not use HC. The other two studies observed 60%^29^ and 80%^28^ increase in CT where women had used UHC and POC, respectively.

The overall estimates of meta-analysis exhibited that HC-use significantly increased the risk of CT in comparison with the control group (pooled effect size by random-effects [reES] = 1.45; 95% CI 1.14–1.84) with the heterogeneity between the studies were 57% (Fig. 2). In the stratified analysis, a statistically significant association was noted between the risk of prevalence of CT and HC-use (reES = 1.58; 95% CI 1.19–2.08) (Fig. 3), but the association was not significant for the risk of incidence of CT (reES = 1.25; 95% CI 0.86–1.82) (Fig. 4).

#### Syphilis/treponema pallidum

Only one study was identified in the group of ST and did not find any significant association with POC use compared to no HC-use or contraceptive use^26^ (Table 1).

#### Trichomonas vaginalis

We found nine studies that analysed the association between HC-use and the risk of incidence or prevalence of TV^21,22,26,27,30–34^ (Table 1). Among those studies, only one study^33^ observed a significant 46% decrease in the risk of TV for POC users.

On the other hand, the meta-analysis demonstrated that HC-use could significantly reduce the risk of TV in comparison to the control group (pooled effect size by fixed-effects [feES] = 0.75; 95%CI:0.65–0.86) (Fig. 2). Interestingly, inter-study heterogeneity was nill. We then separately conducted analyses of the prevalence and incidence of TV with HC-use (Figs. 3 and 4, respectively). Our analysis showed that HC-use significantly reduced the risk of incidence of TV (feES = 0.53; 95% CI 0.35–0.81), but the association was marginally significant for the prevalence of TV (feES = 0.76; 95% CI 0.58–1.00).

#### Pelvic inflammatory disease

Berenson AB, Tan A, Hirth JM and Wilkinson GS ^35^ was the only study that evaluated the association between POC use and PID and found a reduced risk of association (OR = 0.68; 95%CI:0.53–0.86) (Table 1).

#### Herpes simplex virus type 2

Three studies examined the association between HC-use and HSV2 (Table 1) and a significantly increased risk of the acquisition was observed for all^26,36,37^. Two studies observed that POC users were around two times more likely to develop the risk of HSV2^26,37^, and the third one observed that the risk of developing HSV2 was 70% for UHC users^36^.

The overall estimate from the meta-analysis also showed a significant increase in the risk of HSV2 due to the use of HC (pooled effect size by random-effects [reES] = 1.19; 95% CI 1.03–1.37) with the heterogeneity of 44% (Fig. 2). Our stratified analysis reported that HC-use significantly increased the risk of prevalence of HSV2 (feES = 1.86; 95% CI 1.34–2.60) (Fig. 3). The small sample did not allow us to estimate the association between the incidence of HSV2 and HC-use.

#### Bacterial vaginosis

We identified 25 studies that investigated the association of BV with any HC-use. Five studies reported more than one association for the use of different types of HC (Table 1). We observed that a total of nine associations demonstrated a significant decrease in BV for different types of HC-use. For the women who had used COC, three studies^27,38,56^ reported 63%, 71% and 31% reduction in BV, respectively. While among the POC users, a decrease in BV was observed with proportions varying from 34 to 66%^45,48,55,56^. Besides these, two other studies found a protective association with 39%^40^ and 54%^49^, where women had used the UHC.

Our meta-analysis demonstrated that HC-use could significantly reduce the risk of BV in comparison with no HC-use or no contraceptive use (pooled effect size by random-effects [reES] = 0.81; 95% CI 0.76–0.86) with the heterogeneity between the studies were 33% (Fig. 2). The stratified analysis of prevalence and incidence of BV with HC-use (Figs. 3 and 4) also showed a decreased risk of association (reES = 0.68; 95% CI 0.61–0.77 and reES = 0.80; 95% CI 0.67–0.96, respectively).

### Sensitivity analysis

Several sensitivity analyses were carried out to check the influence of certain studies and populations on the overall estimates (e.g., Supplementary Figs. 1 and 2). We did not observe any changes in the direction of the association that was identified through our estimations due to the removal of HIV + studies or sex worker service setting. We also did not observe any major quantitative change in our estimation after the exclusion of those studies except for HSV2. A smaller number of studies in HSV2 group may be the reason for the quantitative change in that group [from 1.19 (Fig. 2) to 2.26 (Supplementary Fig. 2)].

### Risk of bias across studies

**Figure 5:**
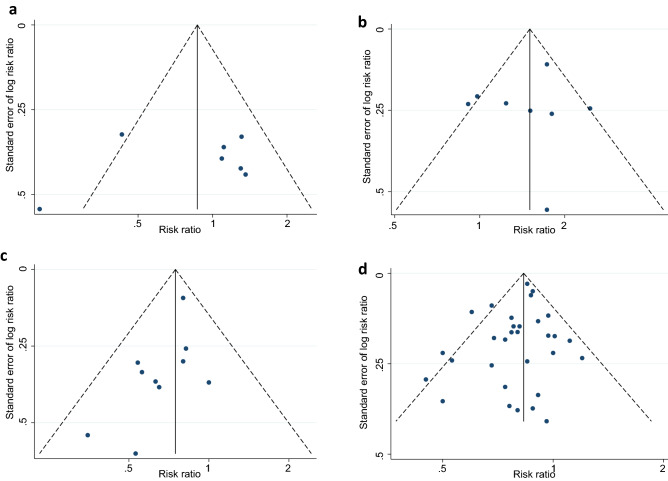
Funnel plots demonstrating the potential presence of publication bias in studies reporting (**a**) Neiserria Gonorrhoea, (**b**) Chlamydia Trachomatis, (**c**) Trichomonas Vaginalis and (**d**) Bacterial Vaginoses. The scatter (in each figure) represents single study.

We conducted funnel plots using prevalent and incident estimates for STIs to explore the heterogeneity in the associations (Fig. 5). The funnel plot of the association between HC-use and the composite outcome of STIs showed an approximately symmetrical distribution with no indication of publication bias (Egger's Bias coefficient = − 0.11, 95% CI − 0.81, 0.60, p = 0.76). The funnel plots for prevalent STIs also indicated an almost symmetrical distribution with the extinction of publication bias (Egger's Bias coefficient = − 0.31, 95% CI − 1.55, 0.93, p = 0.61). The third funnel plots for incident STIs indicated a little asymmetry but we did not observe any significant indication of publication bias (Egger's Bias coefficient = 1.68, 95% CI: − 0.27, 3.63, p = 0.10).

**Figure 6:**
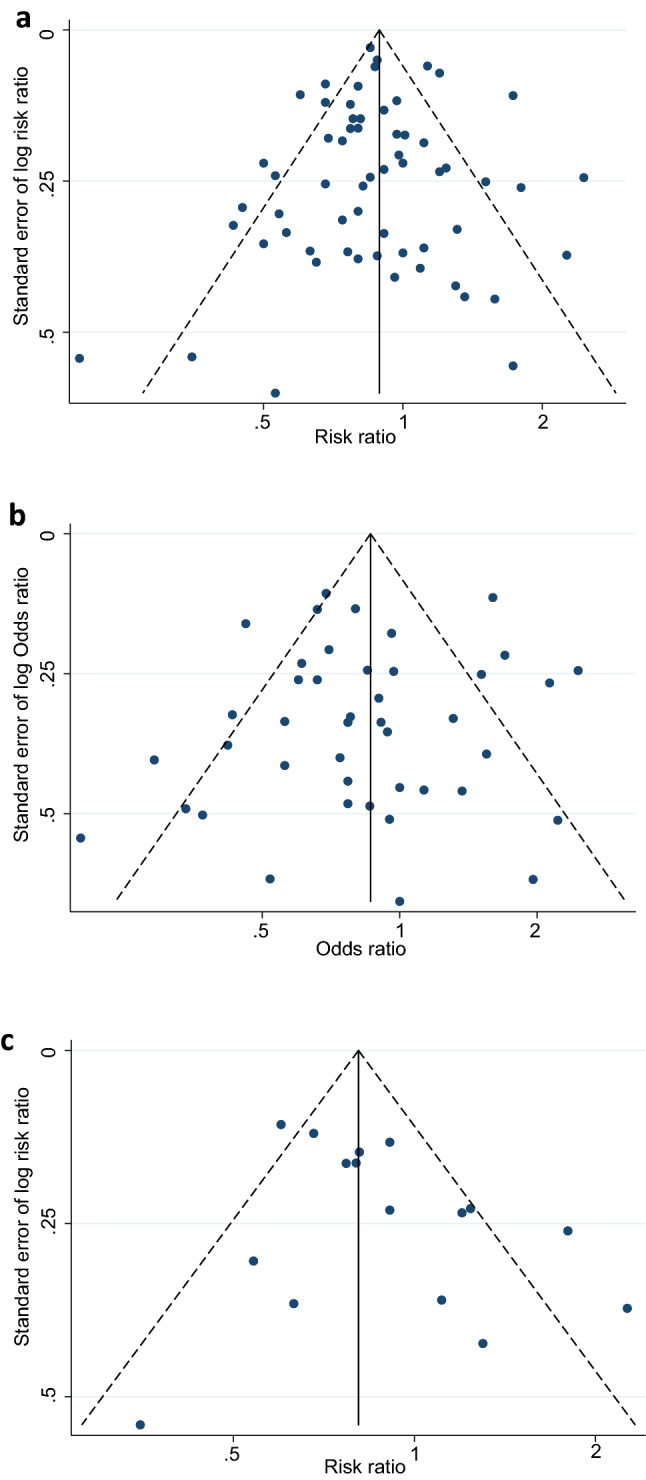
Funnel plots demonstrating the potential presence of publication bias in studies reporting (**a**) composite outcome of any STIs/BV, (**b**) prevalent outcome of any STIs/BV, and (**c**) incident outcome of any STIs/BV. The scatter represents single study.

We also conducted funnel plots using estimates within the individual STIs (Fig. 6). The association of HC-use with CT (Egger's Bias coefficient = − 0.69, 95% CI − 4.21, 2.83, p = 0.65), TV (Egger's Bias coefficient = − 0.83, 95% CI − 1.70, 0.03, p = 0.06), and BV (Egger's Bias coefficient = − 0.48, 95% CI − 1.14, 0.19, p = 0.15) showed an approximately symmetrical distribution with no indication of publication bias. On the other hand, the funnel plots for NG indicated an asymmetry but we did not observe any significant indication of publication bias (Egger's Bias coefficient = − 2.22, 95% CI − 12.92, 8.47, p = 0.62). Note that we did not perform a funnel plot for ST, PID and HSV2 due to the smaller number of studies.

## Discussion

This systematic review and meta-analysis investigated the association between the use of HC and the acquisition of STIs/BV. The findings of this review demonstrated a diverse association when examining the effect of HC-use on the risk of specific STIs/BV. We observed that HC-use increased the risk of CT and HSV2 in comparison with no HC-use or no contraceptive use, but a negative association was observed for TV and BV. We also found a decreased, but statistically insignificant association between HC-use and NG. Unfortunately, a limited number of studies did not allow us to conclude the relationship of HC-use with ST and PID. The diverse relationships between HC-use and different types of STIs can be biological and reflect a complex interaction between the infectious pathogen, the host’s immune response and the vaginal microbiome. This may also be affected by differences in sexual behaviour, testing and clinical care as well as a women's menstrual cycle, length of HC use, ethnicity and geographical locale^45^. Research is needed to identify the exact mechanisms of infections such a CT, NG and BV and HC in the presence of different types of HC, biological and behavioural factors. We discuss our findings in relation to the literature below.

### HC-use and chlamydia trachomatis

The estimates of our meta-analysis indicated that HC-users experienced a significantly increased risk of CT compared to the control group. Two previous reviews^6,7^, observed a similar association, although their control group selection criteria were not comparable with this study. Interestingly, MacCarthy et al.^5^ who conducted a review similar to our investigation, reported an inconclusive association between HC-use and CT. The study, unlike ours, did not use a meta-analysis which can be responsible for the difference in findings. Several biological factors may influence susceptibility to CT infection. For example, exposure to DMPA induces a systemic hypo-estrogenic state associated with decreased vaginal colonization with healthy microbiota, which may increase the risk of CT infection^28^. The anatomical and physiological effects of combined HC may also modulate the pathogenesis of Sexually transmitted diseases (STDs). Users of oral contraceptives, a popular HC, often suffer from cervical ectopy, which may enhance susceptibility to CT^23^. The significant association we found between HC use and CT prevalence but not between HC use and CT incidence may be the result of a small sample size. However, this could also be linked to challenges relating to treating chlamydia infections, as some antibiotic resistant strains may prolong treatment and thus lead to an increase in the prevalence.

However, in contrast to our findings, DMPA users in a study by Deese et al.^59^ were found to have a significantly lower risk of chlamydia compared with the levonorgestrel implant and copper IUD groups. This may be partially explained by research that demonstrates that the initiation of the copper IUD is associated with an increased prevalence of BV and BV is associated with increased rates of CT and NG^60^. Our finding showing that HC-use can significantly increase the risk of CT alongside a significantly reduced risk of BV compared to the control group is not consistent with this research^58^. There may be two explanations for the relationship between DMPA and risk of BV. The prolonged effect of the hormones in DMPA and the absence of a cyclical change in hormonal levels may affect the milieu of the vaginal epithelium, which would make it less favourable for BV to thrive. The other explanation may be due to the constant pH due to the contraceptive effect, which may make it more favourable for less BV.

Several studies have investigated the effects of HC use on immune responses in the female genital tract^34,61^. Research has examined the relationship between DMPA and Norethisterone enanthate use and the impact of immune cell responsiveness to identify whether using these POC may increase susceptibility to infections. However, in a study by Matubu et al.^62^ the exposure of CD4 + and CD8 + T cells to typical pharmacologic concentrations of DMPA was not found to cause immunosuppressive effects. Despite this, the depletion of cytokine-producing T cells may occur after prolonged DMPA use. Length of use may therefore be a factor in exposure to STI risk and could also contribute to the significantly increased risk of CT compared to the control group we identified in our meta-analysis.

### HC-use and trichomonas vaginalis

The reduction in the risk of TV associated with HC-use, observed in our study, was also evident in McCarthy et al.^5^, specifically DMPA and OCP. Mohllajee et al.^6^ and Morrison et al.^7^ could not conclude about the effect of HC-use on TV. As mentioned earlier, both of those studies were dated and their selection criteria were different from ours. A potential reason for this association is that TV requires both oestrogen and androgen receptors. The progesterone receptor is highly expressed in DMPA users or that high MPA concentrations in DMPA users may prevent TV binding to the androgen receptor by competing for receptor binding^33,63^.

### HC-use and herpes simplex virus type 2

We observed a significant increase in the risk of HSV2 due to the use of HC in the meta-analysis, although the estimation was based on a small number of studies. McCarthy et al.^5^ and Deese et al.^10^ also documented a similar association in their review in relation to DMPA use. It is worth mentioning that the control group selection criteria were different in the latter study. A possible biological mechanism was investigated in a recent study on mice^64^. The study demonstrated that DMPA and levonorgestrel, another type of progestin, increase mucosal epithelial permeability by acting on epithelial cell junction proteins, enhancing access to inflammatory and infectious viral molecules to the genital tissue^5,64^.

### HC-use and bacterial vaginosis

Our review concluded that HC-use can significantly reduce the risk of BV that was similar to two previous systematic reviews, Vodstrcil et al.^9^ and Van de Widjert et al.^8^. Examining the association of HC-use in our analysis, separately for the prevalence and the incidence of BV, also showed a negative association. These findings were also consistent with a previous review, Vodstrcil et al.^9^. Menses destabilize the vaginal flora resulting in high concentrations of non-lactobacillus species that favour BV. HC users can experience reduced menses due to use of progestin, reducing BV rates^22,65^. For oestrogen-containing contraceptive users, there is another plausible explanation for experiencing a reduced risk of BV. Such contraceptives increase the glycogen-content of epithelial cells that is metabolised to lactic acid, a suspected primary vaginal acidifier and a known inhibitor of BV^9,66,67^.

### HC-use and Neiserria Gonorrhoea

Our study observed that HC-use did not significantly reduce the risk of NG. Many other reviews also documented an inconclusive association on the acquisition of NG with HC-use^5–7^. However a study by Deese et al. found lower prevalence rates of NG in DMPA users compared with copper intrauterine device^59^. Progesterone-based HC may induce thickening of cervical mucus, limiting STD acquisition, including NG^23^. The reduced incidence of NG can also be due to the reduction in menstrual blood flow for combined HC-users. This is because HC regulation may result in a lighter menstrual flow, which by reducing iron sources, can inhibit the growth of NG since Gonococcal growth is augmented in iron-rich menstrual blood^23,68,69^.

### Limitations

This study has some potential limitations. Firstly, a smaller number of studies did not allow a meta-analysis to be conducted that was stratified by the type of HC. Such analyses are important to better understand the differential effects of COCs and POCs in developing STIs and are more useful for planning public health policies and programmes. Despite this, differences have been found between the effects of types of HCs and STIs in research as noted above for DMPA and CT, TV and NG^5,10,59^.

A limited number of studies also did not allow us to draw a conclusion concerning ST and PID. Secondly, selected studies may suffer from a publication bias because most published studies only report significant results. However, this may not be an issue in our case, as we observed a very low level of publication bias indicated by funnel plots and Egger’s bias tests. Thirdly, the majority of the selected studies in this review provided a prevalence estimate that may indicate only an association between HC and STIs/BV. However, identifying causal effects is more useful for health care decision-making. Fourthly, the control groups were not the same in all studies. Again, this may not be an issue in our case, as sensitivity analyses indicated the robustness of our results. Fifthly, following the previous review^9^, we included studies in which women self-reported HC use was selected. As noted by Pyra et al.^70^, a significant proportion of women self-reporting HC use often had no hormones detected. Though, the sensitivity analysis did not observe any impact on the findings of this research due to the removal of those studies; consequently, it may not be an issue for our research. Finally, we did not perform the adjusted analyses despite the presence of several confounding factors in the current study (e.g. different geographical regions, duration of use of HC). However, the use of adjusted estimates of the selected studies in the meta-analyses reduces the chance of confounding effect on the estimates.

## Conclusion

This systematic review and meta-analysis provide evidence that HC-use influences a woman's risk of STIs/BV, but the risk may differ depending on the type of STIs. We observed a positive association between HC-use and the risk of CT and HSV2 but a negative association for TV and BV. A negative but statistically insignificant association was observed between HC-use and NG. The differences in HC-use and the risk profiles of various types of STIs has clinical implications. Counselling and care for both contraception and infectious disease protection must be provided to all sexually active individuals.

We conclude that there is a need for more studies to investigate the association between different categories of HC and types of STIs. Research is needed to address the lack of knowledge concerning the exact mechanisms of CT, NG and BV infections and HC in the presence of different types of HC, biological and behavioural factors, length of HC use and clinical testing and care. This will reveal further evidence of the complex factors that render individuals more susceptible to STIs according to HC type. Longitudinal cross-sectional or population-based case–control studies are required to estimate causal effects to ensure robust clinical practice and policy formulation. However, rigorously designed studies are needed to enable confounders to be adequately addressed and accounted for. Clinically significant meta-analyses need to be performed to inform guidelines that demonstrate the effect different contraceptives have different effects on STI acquisition. Future research should examine different types of STIs in diverse settings and populations, as a large proportion of previous studies have focused on sex workers and the acquisition of HIV in African contexts.

## Supplementary Information

Supplementary Figure 1.

Supplementary Figure 2.

Supplementary Information 1.

## Data Availability

All available upon request from Tasnima Akter tasnima_akter@yahoo.com.

## Abbreviations

BV: Bacterial vaginosis
COC: Combined oestrogen-progesterone contraception
CRD: Centre for reviews and dissemination
CT: Chlamydia trachomatis
HC: Hormonal contraceptives
HC-use: Hormonal contraceptive use
HIV: Human immunodeficiency virus
HPV: Human papillomavirus
HSV2: Herpes simplex virus type 2
NG: Neisseria Gonorrhoeae
OR: Odds ratio
PID: Pelvic inflammatory disease
PICOS: Population, interventions, comparators, outcomes, study design
POC: Progesterone-only contraception
RCT: Randomised controlled trial
RR: Risk ratio
SE: Standard error
SDG: Sustainable development goal
STD: Sexually transmitted disease
STI: Sexually transmitted infection
ST: Syphilis/treponema pallidum
TV: Trichomonas vaginalis
UHC: Unspecified hormonal contraception
WHO: World Health Organization
