# Supplementary material for: Hormonal contraceptive use and the risk of sexually transmitted infections: a systematic review and meta-analysis
Source: Sci Rep. 2022 Nov 25;12:20325. doi: 10.1038/s41598-022-24601-y (PMC9700818; doi:10.1038/s41598-022-24601-y)
Supplement: Supplementary file 2 — Supplementary Figure 2. [file 41598_2022_24601_MOESM2_ESM.doc]

**Supplementary figure 2: Meta-analysis of the association between hormonal contraceptives and STIs/BV outcome omitting studies with HIV-positive patients**

Note: the diamond in the middle of each horizontal line represents the point estimate of the effect for a single study. Each horizontal lines depict the 95% confidence interval (CI) for a study and the lines that extend beyond the specified value range are cropped and adorned with arrows. The group-specific effect size is plotted by diamond (without horizontal line) with the width corresponding to its 95% CI.
